# HaploVar: an R package for defining local haplotype variants for trait association and trait prediction analyses

**DOI:** 10.1093/bioinformatics/btaf602

**Published:** 2025-12-06

**Authors:** Tessa R MacNish, Hawlader A Al-Mamun, Thomas Bergmann, Mitchell S Bestry, Jacob I Marsh, David Edwards

**Affiliations:** School of Biological Sciences, The University of Western Australia, Perth, WA 6009, Australia; Center for Applied Bioinformatics, The University of Western Australia, Perth, WA 6009, Australia; School of Biological Sciences, The University of Western Australia, Perth, WA 6009, Australia; Center for Applied Bioinformatics, The University of Western Australia, Perth, WA 6009, Australia; InterGrain Pty Ltd, Perth, WA 6163, Australia; School of Biological Sciences, The University of Western Australia, Perth, WA 6009, Australia; Center for Applied Bioinformatics, The University of Western Australia, Perth, WA 6009, Australia; School of Biological Sciences, The University of Western Australia, Perth, WA 6009, Australia; Center for Applied Bioinformatics, The University of Western Australia, Perth, WA 6009, Australia; Department of Biology, University of North Carolina, Chapel Hill, NC 27599, United States; School of Biological Sciences, The University of Western Australia, Perth, WA 6009, Australia; Center for Applied Bioinformatics, The University of Western Australia, Perth, WA 6009, Australia

## Abstract

**Summary:**

Marker assisted breeding (MAB) supports breeding by identifying individuals or molecular markers associated with important traits. MAB methods include genome-wide association studies (GWAS) and genomic selection (GS). Local haplotypes are regions of DNA that are inherited together due to high levels of linkage disequilibrium. Local haplotypes can improve the prediction accuracy and power of GS and GWAS. Currently available local haplotyping tools improve GWAS power through fine-mapping of candidate regions or through haplotype-based GWAS. However, no local haplotyping tools utilize the benefits of haplotypes for GS. Here we present HaploVar, a local haplotyping tool designed to improve both GWAS and GS pipelines by identifying local haplotypes and formatting the output to be compatible with all major GWAS and GS tools. HaploVar can be used in any haplotype-based MAB study.

**Availability and implementation:**

HaploVar can be downloaded from CRAN with R >4.0.0 (DOI: 10.32614/CRAN.package.HaploVar). HaploVar and a tutorial vignette is available on GitHub (https://github.com/TessaMacNish/HaploVar). HaploVar is available under an MIT license.

## 1 Introduction

Marker assisted breeding (MAB) is a crop and animal breeding method that selects individuals by identifying molecular markers associated with important traits. Genome-wide association studies (GWAS) are a MAB method that uses linkage disequilibrium (LD) to associate traits of interest with single nucleotide polymorphisms (SNPs) which can function as molecular markers (Klein *et al.* 2005). GWAS is an effective tool for improving simple monogenic traits, where a few genes have a large effect. These simple traits can be applied through gene pyramiding which stacks the molecular markers identified by GWAS into a single genotype (Servin *et al.* 2004). Improving complex polygenic traits, where there are many genes with a small or varied effect on the trait of interest, is more effective using genomic selection (GS). GS is a MAB method that predicts trait expression in the form of genomic estimated breeding values (GEBVs). These GEBVs can then be used to identify optimal individuals for breeding (Meuwissen *et al.* 2001).

Local haplotypes are multiallelic molecular markers consisting of a group of SNPs that are inherited together due to high levels of linkage disequilibrium (Belzile *et al.* 2020). The use of haplotypes can improve the results of GWAS and GS analyses by accounting for local epistatic effects. Haplotype-based GWAS has greater power to detect QTLs, lower false discovery rate, can map QTLs to a smaller region, and identify a greater number of novel QTLs than conventional SNP-based GWAS (Pryce *et al.* 2010, Chen *et al.* 2021, Helal *et al.* 2021, Wu *et al.* 2022). Haplotypes can improve trait prediction accuracy in GS, especially for traits with high haplotype epistatic heritability (Sallam *et al.* 2020, Bian *et al.* 2021, Weber *et al.* 2023).

There are two main categories of local haplotyping tools, gene-centric and clustering tools. Gene-centric tools define a haplotype as a small region centered around a gene and can use a fixed length to determine the haplotype region around the gene, e.g. Candihap (Li *et al.* 2023) or RFGB (Wang *et al.* 2020), or they can define the haplotype based on LD in the region surrounding the gene, e.g. HaploMiner (Tardivel *et al.* 2019). Clustering local haplotyping tools such as crosshap (Marsh *et al.* 2023) arranges molecular markers into groups based on LD. Crosshap defines local haplotypes in a small region of the genome and visualizes the results. These gene-centric and clustering local haplotyping tools are designed to investigate a small region in greater detail. They are best used once a candidate gene, or a region of interest has been identified and are not suitable for defining genome-wide haplotypes for use in haplotype-based MAB. In contrast, HapFM clusters SNPs into local haplotypes and then performs GWAS (Wu *et al.* 2022). Wu *et al.* (2022) found consistently higher mapping power using HapFM when compared to SNP-based GWAS algorithms, demonstrating how haplotypes can improve MAB results. However, HapFM is limited to GWAS analyses and cannot be used for other MAB approaches.

Here we present HaploVar, a local haplotyping tool that clusters SNPs into LD-based haplotypes, identifies haplotype variants and formats the output to be compatible with common GWAS and GS tools. The purpose of HaploVar is to improve GWAS and GS pipelines by supporting haplotype-based MAB methods.

## 2 Main

HaploVar accepts genotype data in VCF format and a pairwise LD matrix and uses these to define local haplotypes, identify the variants for each haplotype, and to output these local haplotype variants in one of six formats for input into a wide range of GWAS and GS tools. The LD matrix can be calculated using tools such as PLINK (Purcell *et al.* 2007, https://zzz.bwh.harvard.edu/plink/ld.shtml) or the ld function of snpStats (Clayton 2025). HaploVar has two sets of functions: local_haplotypes, which defines local haplotypes and produces a list of local haplotypes and their SNPs; and haplotype_variants, which identifies haplotype variants, and formats the output for GWAS or GS analyses. These function sets are discussed in more detail in sections 2.1 and 2.2, respectively. In each function set there is the base function, a function with the suffix _globally which runs the base function over a list of VCF and corresponding LD matrices, and a function with prefix collate_ which collates output from the base function. These functions are designed to optimize use of computational resources. HaploVar can be installed using the following R code:

install.packages(“HaploVar”)

library(HaploVar)

### 2.1 Defining local haplotypes

The function define_haplotypes uses density-based spatial clustering of applications with noise (DBSCAN) (Ester *et al.* 1996) and a LD matrix to cluster SNPs from the corresponding VCF file into local haplotypes. The benefits of DBSCAN over haplotyping methods include DBSCAN’s ability to scale to large datasets, be robust to outliers, and define clusters of non-spherical or arbitrary shapes which can better represent complex LD patterns (Ester *et al.* 1996, Khvorykh *et al.* 2023). DBSCAN clusters all SNPs considered to be noise into a single group, which is then removed. The parameter keep_outliers = FALSE (default) removes outlier SNPs from within each haplotype. Outliers are removed by calculating the mean LD value for each SNP within the haplotype and removing any SNPs with a mean LD more than 2 standard deviations outside the median intra-haplotype LD value. The parameters epsilon (default = 0.6) and MGmin (default = 30) are used by DBSCAN and influence the densityand amount of noise within the local haplotype. The parameter hetmiss_as determines how missing data is handled for instances where one allele in a genotype is missing (e.g. “./1”). If hetmiss_as = “allele”, the missing allele is assumed to be the reference allele 0 by default. If hetmiss_as = “miss”, the genotype is treated as NA. The function define_haplotypes produces a list of tables. Each table represents one local haplotype and contains the genotypes for all SNPs within that haplotype. Haplotypes are named according to the position of their first and last SNPs (e.g. “hap_31604185_31646449”).

The function define_haplotypes_globally runs define_haplotypes for a list of VCF files and corresponding LD matrices and collates the results into one list of local haplotype tables. The final function for defining local haplotypes collate_define_haplotypes requires a list of outputs from define_haplotypes or define_haplotypes_globally and collates the results to form one list of local haplotype tables. The HaploVar package includes two small datasets—vcf and LD—which can be used to run all HaploVar functions. vcf is s subset of *Brassica napus* data published by Wu and colleagues (2019) and downloaded from CropGS-Hub (Chen *et al.* 2024, https://iagr.genomics.cn/CropGS/#/Datasets? species=Rapeseed). LD was generated using PLINK and the parameters—allow-extra-chr and—r2 square (Purcell *et al.* 2007, https://zzz.bwh.harvard.edu/plink/ld.shtml). An example of how to run the base define_haplotypes function is given below:

define_haplotypes(vcf, LD, epsilon = 0.6, MGmin = 30, hetmiss_as =“allele”, keep_outliers = FALSE)

### 2.2 Haplotype variants and formatting

**Figure 1.**
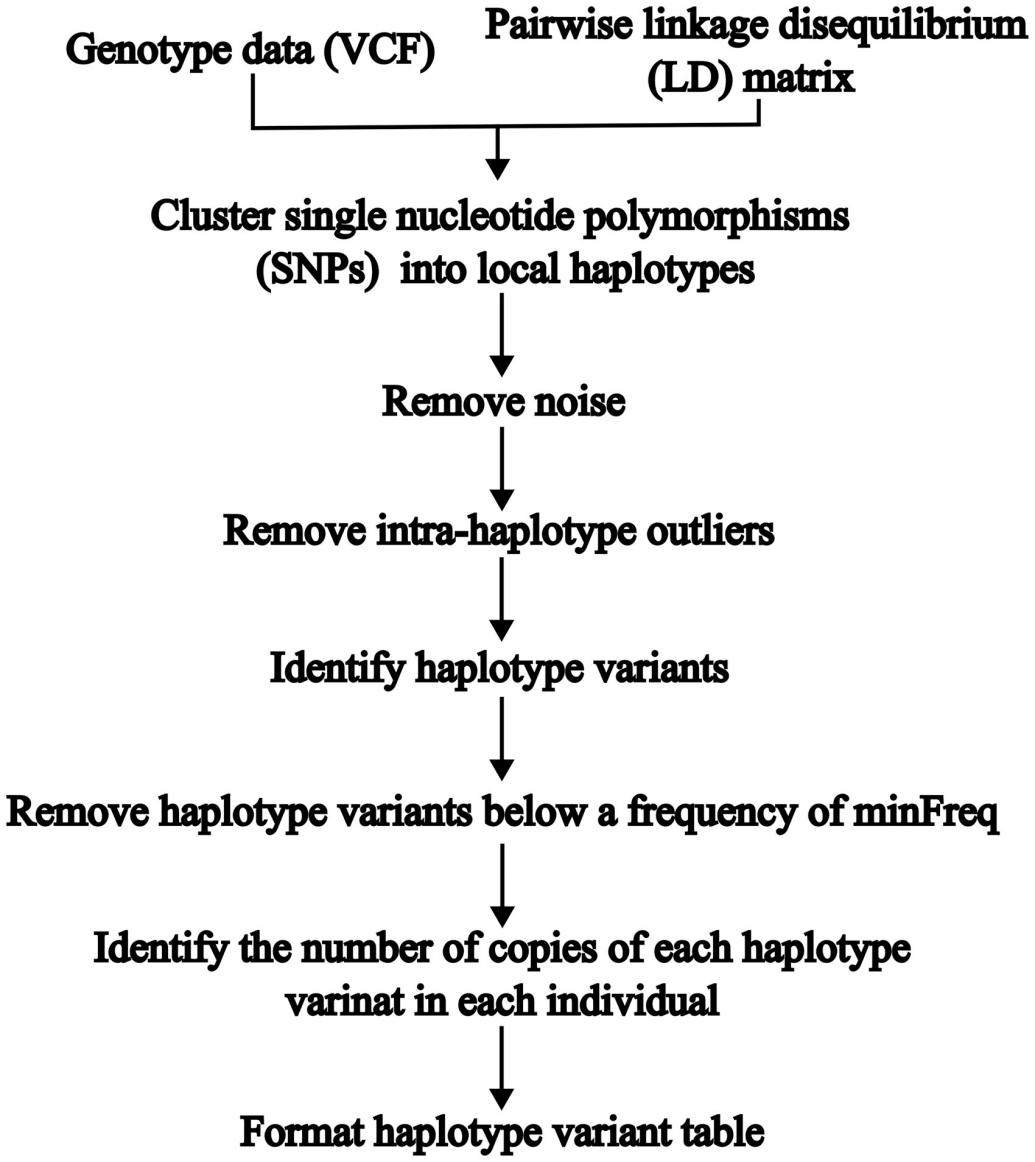
Workflow of HaploVar’s haplotype_variants function.

The function haplotype_variants defines haplotypes and removes outliers in the same way as define_haplotypes. Once the local haplotypes have been defined, haplotype_variants identifies unique combinations of SNPs within each haplotype. Each unique combination of SNPs is a haplotype variant and is allocated a letter (A-Z), or a combination of letters if there are >26 variants within a haplotype (AA-ZZ, with the exclusion of NA). Haplotype variants present in fewer individuals than minFreq (default = 2) are removed. The genotype for individuals where none of the remaining haplotype variants are present are set to 0. The function haplotype_variants outputs one of six formats depending on the parameter format (default = 1) (see Note 1, available as supplementary data at *Bioinformatics* online). The different output tables can be used as input for a wide range of GWAS and GS tools. The general workflow of the haplotype_variants function is shown in Fig. 1. The function haplotype_variants_global runs haplotype_variants for a list of VCF files and corresponding LD matrices and collates the results into one table. The final function for identifying haplotype variants collate_haplotype_variants requires a list of outputs from haplotype_variants or haplotype_variants_global and collates the results to form one table. All tables within the list of output tables must be formatted in the same way. An example of how to run the base haplotype_variants function is given below:

haplotype_variants(vcf, LD, epsilon = 0.6, MGmin = 30, minFreq = 2, hetmiss_as = “ allele ” , keep_outliers = FALSE, format = 1)

Vignettes and documentation on how to use HaploVar are available at https://github.com/TessaMacNish/HaploVar.

## 3 Conclusions and perspectives

HaploVar is designed to improve MAB pipelines by the preparation of haplotype-based data for GWAS and GS. HaploVar takes SNP and LD data, calculates local haplotypes, identifies haplotype variants, and formats the output for use in GWAS and GS tools. An example application of HaploVar can be found in Note 2, available as supplementary data at *Bioinformatics* online. Comparison of HaploVar with other haplotyping tools is shown in Note 3, available as supplementary data at *Bioinformatics* online. Note 4, available as supplementary data at *Bioinformatics* online shows a parameter sensitivity analysis. Using haplotypes in MAB studies can improve power and trait prediction accuracy.

Current local haplotyping tools focus on the downstream analysis and fine-mapping of GWAS results or are limited to only haplotype-based GWAS. HaploVar is the first tool designed utilize the benefits of local haplotypes for both haplotype-based GWAS and haplotype-based GS.

## Supplementary Material

btaf602_Supplementary_Data

## Data Availability

The data underlying this article are available in CropGS-Hub at https://iagr.genomics.cn/CropGS/#/Datasets?species=Rapeseed
