# Supplementary material for: HaploVar: an R package for defining local haplotype variants for trait association and trait prediction analyses
Source: Bioinformatics. 2025 Dec 6;41(12):btaf602. doi: 10.1093/bioinformatics/btaf602 (PMC12684708; doi:10.1093/bioinformatics/btaf602)
Supplement: btaf602_Supplementary_Data [file btaf602_supplementary_data.zip › supplemental material - Second revision.docx]

Supplementary Material for HaploVar: An R package for defining local haplotype variants for trait association and trait prediction analyses

Tessa R. MacNish^1,2^, Hawlader A. Al-Mamun^1,2,3^, Thomas Bergmann^1,2^, Mitchell S. Bestry^1,2^, Jacob I. Marsh^4^, and David Edwards^1,2,*^

^1^School of Biological Sciences, The University of Western Australia, Perth, WA, 6009, Australia.,

^2^Center for Applied Bioinformatics, The University of Western Australia, Perth, WA, 6009, Australia.,

^3^InterGrain Pty Ltd, Perth, WA, 6163, Australia,
^4^Department of Biology, University of North Carolina, Chapel Hill, NC, 27599, USA

*Corresponding author: David Edwards, School of Biological Sciences, The University of Western Australia, Perth

# Supplementary Note 1: Output formats of haplotype_variants

The haplotype variant tables displayed in Supplementary Note 1 are examples of the different formats that HaploVar can output. Each table represents a small subset of the data and is designed to display the relevant column and row structure of each format. All haplotype variant tables were constructed using an epsilon value of 0.8. All other HaploVar parameters, except for format, were set to default. The data used to generate these tables was a subset of a *Brassica napus* genotype dataset published by Wu and colleagues (2019) and downloaded from CropGS-Hub (Chen *et al*., 2024, https://iagr.genomics.cn/CropGS/#/Datasets?species=Rapeseed).

**Supplemental Table 1.** HaploVar’s haplotype_variants output format 1. The first three columns are MARKER, CHROM, and POS, which display the haplotype ID, the chromosome where the haplotype is present, and the position of the first single nucleotide polymorphism (SNP) in each haplotype, respectively. The following columns represent the genotypes for all individuals in the population. The haplotype variants for each haplotype, are represented by letters A to ZZ. Zero represents the absence of any haplotype variant, which may be due to missing data or because there were no valid haplotypes in that region for that individual. This format is designed to give an overview of the variants present for each haplotype.

| **MARKER** | **CHROM** | **POS** | **R4155_R4155** | **R4156_R4156** | **R4157_R4157** | **R4158_R4158** | **R4159_R4159** | **R4160_R4160** |
| --- | --- | --- | --- | --- | --- | --- | --- | --- |
| hap_386610_407953 | chrC01 | 386610 | A\|A | 0\|0 | A\|A | A\|A | A\|A | 0\|0 |
| hap_667407_678097 | chrC01 | 667407 | B\|B | 0\|B | B\|B | BA\|BA | 0\|K | 0\|0 |
| hap_703723_721842 | chrC01 | 703723 | LA\|LA | 0\|0 | LA\|LA | 0\|LA | 0\|0 | S\|O |
| hap_850089_912143 | chrC01 | 850089 | A\|A | A\|A | A\|A | A\|A | A\|A | A\|A |
| hap_886749_913778 | chrC01 | 886749 | A\|A | A\|A | A\|A | A\|A | A\|A | A\|A |

**Supplemental Table 2.** HaploVar’s haplotype_variants output format 2. The first three columns are MARKER, CHROM, and POS, which display the haplotype variant ID, the chromosome where the haplotype variant is present, and the position of the first single nucleotide polymorphism (SNP) in each haplotype variant, respectively. The following columns represent the genotypes for all individuals in the population. Each haplotype variant can be absent from that individual (0), have one copy present (1) or two copies present (2). This format is designed for genome-wide association studies.

| **MARKER** | **CHROM** | **POS** | **R4155_R4155** | **R4156_R4156** | **R4157_R4157** | **R4158_R4158** | **R4159_R4159** | **R4160_R4160** |
| --- | --- | --- | --- | --- | --- | --- | --- | --- |
| hap_386610_407953_A | chrC01 | 386610 | 2 | 0 | 2 | 2 | 2 | 0 |
| hap_386610_407953_B | chrC01 | 386610 | 0 | 0 | 0 | 0 | 0 | 0 |
| hap_386610_407953_K | chrC01 | 386610 | 0 | 0 | 0 | 0 | 0 | 0 |
| hap_386610_407953_R | chrC01 | 386610 | 0 | 0 | 0 | 0 | 0 | 0 |
| hap_386610_407953_F | chrC01 | 386610 | 0 | 0 | 0 | 0 | 0 | 0 |
| hap_386610_407953_G | chrC01 | 386610 | 0 | 0 | 0 | 0 | 0 | 0 |
| hap_386610_407953_D | chrC01 | 386610 | 0 | 0 | 0 | 0 | 0 | 0 |

**Supplemental Table 3.** HaploVar’s haplotype_variants output format 3. This haplotype variant table is a matrix of n$\times$m where n is the number of individuals in the population and m is the number of haplotype variants. Each haplotype variant can be absent from that individual (0), have one copy present (1) or two copies present (2). This format is designed for genomic selection studies in a package such as STGS (Budhlakoti *et al*. 2019).

|  | **hap_386610_407953_A** | **hap_386610_407953_B** | **hap_386610_407953_K** |
| --- | --- | --- | --- |
| **R4155_R4155** | 2 | 0 | 0 |
| **R4156_R4156** | 0 | 0 | 0 |
| **R4157_R4157** | 2 | 0 | 0 |
| **R4158_R4158** | 2 | 0 | 0 |
| **R4159_R4159** | 2 | 0 | 0 |
| **R4160_R4160** | 0 | 0 | 0 |

**Supplemental Table 4.** HaploVar’s haplotype_variants output format 4. The first three columns are MARKER, CHROM, and POS, which display the haplotype variant ID, the chromosome where the haplotype variant is present, and the position of the first single nucleotide polymorphism (SNP) in each haplotype variant, respectively. The following columns represent the genotypes for all individuals in the population. Each haplotype variant can be absent from that individual (-1), have one copy present (0) or two copies present (1). This format is designed for genome-wide association studies using a package such as rrBLUP (Endelman, 2011).

| **MARKER** | **CHROM** | **POS** | **R4155_R4155** | **R4156_R4156** | **R4157_R4157** | **R4158_R4158** | **R4159_R4159** | **R4160_R4160** |
| --- | --- | --- | --- | --- | --- | --- | --- | --- |
| hap_386610_407953_A | chrC01 | 386610 | 1 | -1 | 1 | 1 | 1 | -1 |
| hap_386610_407953_B | chrC01 | 386610 | -1 | -1 | -1 | -1 | -1 | -1 |
| hap_386610_407953_K | chrC01 | 386610 | -1 | -1 | -1 | -1 | -1 | -1 |
| hap_386610_407953_R | chrC01 | 386610 | -1 | -1 | -1 | -1 | -1 | -1 |
| hap_386610_407953_F | chrC01 | 386610 | -1 | -1 | -1 | -1 | -1 | -1 |
| hap_386610_407953_G | chrC01 | 386610 | -1 | -1 | -1 | -1 | -1 | -1 |
| hap_386610_407953_D | chrC01 | 386610 | -1 | -1 | -1 | -1 | -1 | -1 |

**Supplemental Table 5.** HaploVar’s haplotype_variants output format 5. This haplotype variant table is a matrix of n$\times$m where n is the number of individuals in the population and m is the number of haplotype variants. Each haplotype variant can be absent from that individual (-1), have one copy present (0) or two copies present (1). This format is designed for genomic selection studies using a package such as rrBLUP (Endelman, 2011).

|  | **hap_386610_407953_A** | **hap_386610_407953_B** | **hap_386610_407953_K** |
| --- | --- | --- | --- |
| **R4155_R4155** | 1 | -1 | -1 |
| **R4156_R4156** | -1 | -1 | -1 |
| **R4157_R4157** | 1 | -1 | -1 |
| **R4158_R4158** | 1 | -1 | -1 |
| **R4159_R4159** | 1 | -1 | -1 |
| **R4160_R4160** | -1 | -1 | -1 |

**Supplemental Table 6.** HaploVar’s haplotype_variants output format 6. Format 6 outputs a VCF file and is designed for genome-wide association studies and can be used within tools such as PLINK (Purcell et al. 2007, https://zzz.bwh.harvard.edu/plink/ld.shtml) or GEMMA (Zhou and Stephens, 2012).

| **CHROM** | **POS** | **ID** | **REF** | **ALT** | **QUAL** | **FILTER** | **INFO** | **FORMAT** | **R4155_ R4155** | **R4156_ R4156** |
| --- | --- | --- | --- | --- | --- | --- | --- | --- | --- | --- |
| chrC01 | 386610 | hap_386610_407953_A | . | . | . | PASS | . | GT | 1\|1 | 0\|0 |
| chrC01 | 386610 | hap_386610_407953_B | . | . | . | PASS | . | GT | 0\|0 | 0\|0 |
| chrC01 | 386610 | hap_386610_407953_K | . | . | . | PASS | . | GT | 0\|0 | 0\|0 |
| chrC01 | 386610 | hap_386610_407953_F | . | . | . | PASS | . | GT | 0\|0 | 0\|0 |
| chrC01 | 386610 | hap_386610_407953_G | . | . | . | PASS | . | GT | 0\|0 | 0\|0 |
| chrC01 | 386610 | hap_386610_407953_D | . | . | . | PASS | . | GT | 0\|0 | 0\|0 |
| chrC01 | 386610 | hap_386610_407953_I | . | . | . | PASS | . | GT | 0\|0 | 0\|0 |
| chrC01 | 386610 | hap_386610_407953_C | . | . | . | PASS | . | GT | 0\|0 | 0\|0 |

# Supplementary Note 2: Example application of HaploVar - Haplotype-based GWAS for flowering time in *Brassica napus*

In this section we demonstrate the use of HaploVar for a genome-wide association study (GWAS). The GWAS analysis was performed in *Brassica napus* for flowering time.

## S2.1 Methods

The *B. napus* data published by Wu and colleagues (2019) and downloaded from CropGS-Hub (Chen *et al.,* 2024, https://iagr.genomics.cn/CropGS/#/Datasets?species=Rapeseed), includes single nucleotide polymorphism (SNP) data in VCF format and covariate data including the country of origin and the ecotype (spring, winter and semi-winter) for 991 individuals. The VCF file contains 4,286,896 SNPs. The number of days to flowering was measured for 926 of the 991 individuals (Wu *et al.* 2019). The VCF file was filtered for minor allele frequency (MAF) (--maf 0.05), minimum depth (-‑minDP 5), and maximum missingness (--max-missing 0.5), using VCFtools version 0.1.16 (Danecek *et al.* 2011). All insertions and deletions (indels) were removed from the VCF file (--remove-indels). After filtering the VCF contained 2,222,720 SNPs. The filtered VCF was phased and imputed using Beagle version 5.4 with the default settings (Browning *et al*. 2018; Browning *et al.* 2021). The filtered, phased and imputed VCF was separated by chromosome using BCFtools version 1.15 (Danecek et al., 2021) and the view function with default settings. The chromosome VCFs were further split into four segments each. PLINK generated pairwise linkage disequilibrium matrices using the parameters –allow-extra-chr and –r2 square (Purcell *et al.* 2007, https://zzz.bwh.harvard.edu/plink/ld.shtml) for each chromosome segment VCF. The filtered, phased, and imputed chromosome segment VCFs and their corresponding linkage disequilibrium matrices were input into Haplovar’s haplotype_variants function. The epsilon was set to 0.7, the format was set to 6 and all other HaploVar parameters were set to default. The haplotype tables for each chromosome segment were input into Haplovar’s collate_haplotype_variants function with format set to 6. The resulting haplotype variant VCF, with a total of 984 haplotype variants, was used in the GWAS analysis.

Flowering time values more than three standard deviations from the mean were considered outliers and were removed from the dataset. After outliers were removed, flowering time data for 918 individuals remained. A linear model was calculated in R (R Core Team, 2024) using flowering time, country of origin, and ecotype data. The residuals from the linear model were used as the phenotype data in the GWAS. The following code was used to generate the linear model and residuals:

model1 = lm(data$FloweringTime~ data$Ecotype + data$Country)

residuals1 = model1$residuals

The GWAS was conducted using GEMMA’s linear mixed model with the parameter -maf 0 and all other parameters set to default (Zhou and Stephens, 2012). The p-values were corrected using false discovery rate (FDR) (Benjamini and Hochberg, 1995), using the following R code:

FTresultGemma0.7_MGmin_default_minFreq_default <- read_table("FT_H0.7.lmm.assoc.txt")

FTresultGemma0.7_MGmin_default_minFreq_default$fdrs <-p.adjust(FTresultGemma0.7_MGmin_default_minFreq_default$p_lrt, method="BH")

The quantile-quantile and Manhattan plots were created using the R package qqman (Turner, 2018) and the following code:

FTresultGemma0.7_MGmin_default_minFreq_9_manhattan <- manhattan(FTresultGemma0.7_MGmin_default_minFreq_default,chr="chr",bp="ps",p="fdrs",snp="rs",genomewideline=-log10(0.05), suggestiveline = F, highlight = "hap_28471579_28613643_S")

FTresultGemma0.7_MGmin_default_minFreq_default_qq <- qq(FTresultGemma0.7_MGmin_default_minFreq_default$p_lrt)

BLAST version 2.12.0 (Altschul *et al*. 1990) was used to identify the locations of the *FLOWERING LOCUS T* (*FT*) and *FLOWERING LOCUS C* (*FLC)* orthologs in the *B. napus* dataset. An example of the BLAST code for the *FT* orthologs is as follows:

makeblastdb -in Brassica_napus_v4.1.chromosomes.fasta -parse_seqids -dbtype nucl -out B_napus_DB

blastn -query FT.fasta -db B_napus_DB -out FT_vs_B_napus_cropGS_BLAST.out -evalue 1e-10 -outfmt "6 qseqid sseqid pident length mismatch gapopen qstart qend sstart send evalue bitscore qseq sseq" -num_threads 64

## S2.2 Results and Discussion

The quantile-quantile and Manhattan plots are displayed in Supplemental Figures 1 and 2. None of the significantly associated haplotype variants were near a *FLC* ortholog. *FT* ortholog *BnaC06g27090D* was found to be located at 28555216 bp to 28552966 bp on chromosome C06. This *FT* ortholog is located within the significantly associated haplotype variant hap_28471579_28613643_S, with the start position of 28471579 bp and the end position of 28613643 bp. Haplotype variant hap_28471579_28613643_S was the third most significant GWAS result. The identification of a known flowering gene within a significant haplotype demonstrates the ability of HaploVar to identify true associations in a GWAS analysis. The flowering time GWAS conducted by Wu *et al*. (2019) identified SNPs associated with the *FT* ortholog *BnaA02g12130D* on chromosome A02 and the *FLC* ortholog *BnaA10g22080D* on chromosome A10. These findings are consistent with other GWAS studies that found that SNP-based and haplotype‑based GWAS that used the same dataset, identified unique genomic regions associated with the trait of interest (Abed and Belzile 2019, Bekele *et al*. 2018, Contreras-Soto *et al*. 2017). SNP and haplotype-based GWAS identify different subsets of genomic variation, with haplotype-based GWAS able to identify significantly associated genomic regions that were missed by using SNPs alone (Helal *et al*. 2021, Khvorykh *et al*. 2023). SNPs and haplotypes can be used together to find more causal genetic variants associated with agronomically important traits. In addition, the multiallelic nature of haplotypes allows breeders to remove deleterious variants and select for beneficial ones by identifying which haplotype variants have optimal phenotypic combinations (Clot *et al.* 2024, Meena *et al.* 2025). SNP and haplotype-based GWAS are complementary methods that should be used together to maximize genetic gain and diversity in crops.

| 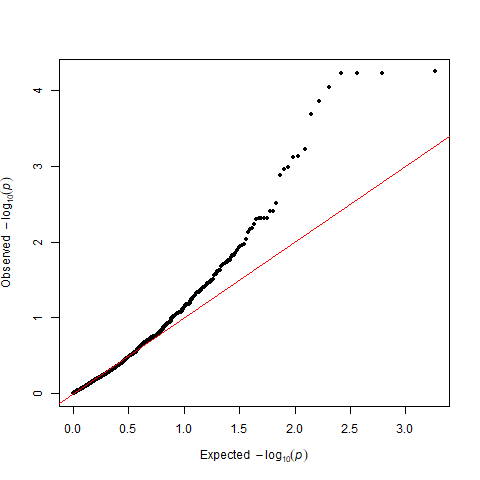 |
| --- |

**Supplemental Figure 1.** A quantile-quantile plot of flowering time in Brassica napus. The SNPs were filtered using VCFtools (Danecek et al. 2011) and the following parameters ‑‑max-missing 0.5 --minDP 5 --maf 0.05 --recode --remove-indels. The SNPs were then phased and imputed using Beagle (Browning et al. 2018; Browning et al. 2021). The SNPs were haplotyped using HaploVar with an epsilon of 0.7. GWAS analysis was done using GEMMA and the parameter -maf 0 (Zhou and Stephens, 2012).

| 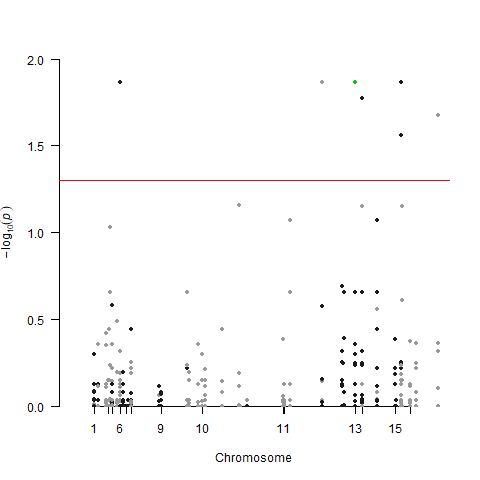 |
| --- |

**Supplemental Figure 2.** A Manhattan plot of flowering time in Brassica napus. The SNPs were filtered using VCFtools (Danecek et al. 2011) and the following parameters -‑max-missing 0.5 --minDP 5 --maf 0.05 --recode --remove-indels. The SNPs were then phased and imputed using Beagle (Browning et al. 2018; Browning et al. 2021). The SNPs were haplotyped using HaploVar with an epsilon of 0.7. GWAS analysis was done using GEMMA and the parameter -maf 0 (Zhou and Stephens, 2012). The p‑values were corrected using false discovery rate (FDR) (Benjamini and Hochberg 1995). The significance threshold (red line) was set to a p-value of 0.05. The haplotype hap_28471579_28613643_S (green dot) contains FLOWERING LOCUS T (FT) ortholog BnaC06g27090D.

# Supplementary Note 3: Comparison of haplotyping tools

In this section we compare the haplotyping tools HaploVar, PLINK, and HaploBlocker. We compare each tool’s computational efficiency, functionality, and the performance of each tool’s haplotyping algorithms in genome-wide association studies (GWAS) and genomic selection (GS), respectively.

S3.1 Methods

### S3.1.1 SNP preparation

The *B. napus* data published by Wu and colleagues (2019) and downloaded from CropGS-Hub (Chen *et al*., 2024, https://iagr.genomics.cn/CropGS/#/Datasets?species=Rapeseed), includes single nucleotide polymorphism (SNP) data in VCF format and covariate data including the country of origin and the ecotype (spring, winter and semi-winter) for 991 individuals. The VCF file contains 4,286,896 SNPs. The number of days to flowering was measured for 926 of the 991 individuals (Wu *et al.* 2019). The VCF file was filtered for minor allele frequency (MAF) (--maf 0.05), minimum depth (-‑minDP 5), and maximum missingness (--max-missing 0.5), using VCFtools version 0.1.16 (Danecek *et al.* 2011). All insertions and deletions (indels) were removed from the VCF file (--remove-indels). After filtering the VCF contained 2,222,720 SNPs. The filtered VCF was phased and imputed using Beagle version 5.4 with the default settings (Browning *et al*. 2018; Browning *et al.* 2021). The filtered, phased and imputed VCF was separated by chromosome using BCFtools version 1.15 (Danecek et al., 2021) and the view function with default settings. The chromosome VCFs were further split into four segments each. PLINK generated pairwise linkage disequilibrium matrices using the parameters --allow-extra-chr and --r2 square (Purcell *et al.* 2007, https://zzz.bwh.harvard.edu/plink/ld.shtml) for each chromosome segment VCF.

### S3.1.2 Phenotype preparation

For all GWAS analyses flowering time values more than three standard deviations from the mean were considered outliers and were removed from the dataset. After outliers were removed, flowering time data for 918 individuals remained. A linear model was calculated in R (R Core Team, 2024) using flowering time, country of origin, and ecotype data. The residuals from the linear model were used as the phenotype data in the GWAS analyses. The following code was used to generate the linear model and residuals:

model1 = lm(data$FloweringTime~ data$Ecotype + data$Country)

residuals1 = model1$residuals

The raw flowering time data, for 926 individuals, was used as the phenotype data in the GS models.

### S3.1.3 HaploVar haplotypes

HaploVar’s haplotype variants were calculated using the haplotype_variants function and the parameters epsilon=0.7, MGmin=10, and minFreq=9. Haplotype variants were calculated for each chromosome segment and were collated using the collate_haplotype_variants function. A VCF for the GWAS analyses was generated using format 6, while format 5 was used to prepare the data for GS.

### S3.1.4 PLINK haplotypes

Haplotype block boundaries were calculated for each chromosome segment using PLINK (Purcell *et al*. 2007, <https://zzz.bwh.harvard.edu/plink/ld.shtml>) and the following parameters --allow-extra-chr and --blocks no-pheno-req. PLINK estimates haplotype blocks using Gabriel’s algorithm (Gabriel *et al*., 2002). The wall time and memory used by PLINK were measured using the sacct (slurm 23.11.10) command. An example of the custom R code was used to identify haplotype variants and format the output for GWAS and GS analyses is shown below:

det_file <- "chrA01_A1_phased_haplotype.blocks.det"

det <- read.table(det_file, header = TRUE, sep = "", stringsAsFactors = FALSE) %>%

as_tibble() %>%

rename(chr = CHR,

start = BP1,

end = BP2,

snp_ids = SNPS)

haplotype_df <- det %>%

mutate(Haplotype = paste0("hap_", start, "_", end)) %>%

select(Haplotype, chr, start, end)

source("haplotype_from_regions.R")

format6 <- haplotype_variants_from_regions(vcf, haplotype_df, minFreq = 9, hetmiss_as = "allele", format = 6)

format5 <- haplotype_variants_from_regions(vcf, haplotype_df, minFreq = 9, hetmiss_as = "allele", format = 5)

The R script haplotype_from_regions.R is available on GitHub (<https://github.com/TessaMacNish/haplotype_variants_from_regions/tree/main>) and zenodo (DOI: https://doi.org/10.5281/zenodo.17292563). The haplotype variant tables were collated using HaploVar’s collate_haplotype_variants function. The haplotype variant table in format 6 was used for GWAS, while the format 5 haplotype variant table was used for GS. The parameter minFreq was set to 9, to be consistent with the methods used to identify haplotype variants using HaploVar.

### S3.1.5 HaploBlocker haplotypes

HaploBlocker’s (version 4.2.3) block_calculation function and default parameters were used to calculate haplotype block boundaries (Pook *et al*., 2019). The wall time and memory used by HaploBlocker were measured using the sacct (slurm 23.11.10) command. An example of the custom R code was used to identify haplotype variants and format the output for GWAS and GS analyses is shown below:

haplotype_info <- list()

all_objs <- ls(pattern = "^result_chr")

for (obj_name in all_objs) {

hap_list <- get(obj_name)

chr <- sub("result_(chr[^_]+)_.*", "\\1", obj_name)

for (hap in hap_list) {

start_bp <- hap[[2]][[3]]

end_bp <- hap[[3]][[3]]

hap_id <- paste0("hap_", start_bp, "_", end_bp)

haplotype_info[[length(haplotype_info) + 1]] <- list(

Haplotype = hap_id,

chr = chr,

start = start_bp,

end = end_bp

)

}

}

haplotype_df <- do.call(rbind, lapply(haplotype_info, as.data.frame))

source("haplotype_from_regions.R")

format6 <- haplotype_variants_from_regions(vcf, haplotype_df, minFreq = 9, hetmiss_as = "allele", format = 6)

format5 <- haplotype_variants_from_regions(vcf, haplotype_df, minFreq = 9, hetmiss_as = "allele", format = 5)

The R script haplotype_from_regions.R is available on GitHub (<https://github.com/TessaMacNish/haplotype_variants_from_regions/tree/main>) and zenodo (DOI: https://doi.org/10.5281/zenodo.17292563). The haplotype variant tables were collated using HaploVar’s collate_haplotype_variants function. The haplotype variant table in format 6 was used for GWAS, while the format 5 haplotype variant table was used for GS.

### S3.1.6 Genome-wide association studies

GWAS was conducted for all three haplotyping methods using GEMMA’s linear mixed model with the parameter -maf 0 and all other parameters set to default (Zhou and Stephens, 2012). The p-values were corrected using false discovery rate (FDR) (Benjamini and Hochberg, 1995). The time and memory used were measured using the sacct (slurm 23.11.10) command.

### S3.1.7 Genomic selection

Genomic best linear unbiased prediction (GBLUP) was run for each haplotyping method using rrBLUP version 4.6.1 (Endelman, 2011). 4-fold cross-validation was performed using caret (Kuhn, 2008) to divide the dataset into 4 equal subsets. 4-fold cross-validation GBLUP was repeated five times for each haplotyping tool with five different random seeds (111, 222, 333, 444, 555). Trait prediction accuracy was calculated using Metrics version 0.1.4 (Hamner & Frasco, 2018) for mean squared error (MSE) and root mean squared error (RMSE). Base R was used to calculate Pearson’s correlation coefficient. All R code was run using R version 4.0.3 (R Core Team, 2024). An example of the GBLUP code is given below:

library(rrBLUP)

library(Metrics)

library(caret)

#Load genotype

GBLUP_PLINK <- read.csv("PLINK.csv", header = TRUE, row.names = 1)

#Load phenotype

Pheno <- read.table("GSTP013.pheno.nospace.txt", header = TRUE, row.names = 1)

#Load covariate

Covar <-read.csv("GSTP013_covariate.csv", header = TRUE)

rownames(Covar) <- rownames(GBLUP_PLINK)

FT_ID <- read.csv("FT_ID.csv", header = TRUE, row.names = 1)

FT_ID$x <- paste0(FT_ID$x, "_", FT_ID$x)

rownames(Pheno) <- paste0(rownames(Pheno), "_", rownames(Pheno))

Pheno_FT <- Pheno[, 1, drop = FALSE]

Pheno_FT <- Pheno_FT[rownames(Pheno_FT) %in% FT_ID$x, , drop = FALSE]

GBLUP_PLINK<- GBLUP_PLINK [rownames(GBLUP_PLINK) %in% FT_ID$x, ]

GBLUP_PLINK_FT <- GBLUP_PLINK [rownames(GBLUP_PLINK) %in% FT_ID$x, ]

Covar_FT <- Covar[rownames(Covar) %in% FT_ID$x, ]

# Combine phenotype and covariates

pheno_gblup <- data.frame(gid = rownames(Pheno_FT), trait = Pheno_FT[[1]], Covar_FT)

# Compute additive relationship matrix (VanRaden method)

K <- A.mat(GBLUP_PLINK_FT)

# Setup 4-fold cross validation

set.seed(222)

folds <- createFolds(pheno_gblup$trait, k = 4, list = TRUE, returnTrain = FALSE)

#Storage for results

mae_list <- c()

rmse_list <- c()

cor_list <- c()

# Run GBLUP

for (i in 1:4) {

cat("Running fold", i, "...\n")

test_idx <- folds[[i]]

pheno_temp <- pheno_gblup

pheno_temp$trait[test_idx] <- NA

rr_model <- kin.blup(data = pheno_temp, geno = "gid", pheno = "trait", K = K, fixed = c("Group", "Country") )

# Extract predictions for the masked individuals

preds <- rr_model$pred[test_idx]

obs <- pheno_gblup$trait[test_idx]

# Save Metrics

mae_val <- mae(obs, preds)

rmse_val <- rmse(obs, preds)

cor_val <- cor(obs, preds, method = "pearson")

mae_list <- c(mae_list, mae_val)

rmse_list <- c(rmse_list, rmse_val)

cor_list <- c(cor_list, cor_val)

}

#Save all fold results

results_summary <- data.frame(

Fold = 1:4,

MAE = mae_list,

RMSE = rmse_list,

Pearson_r = cor_list

)

The time and memory used were measured using the sacct (slurm 23.11.10) command. ANOVA was used to compare the trait prediction accuracy between the three haplotyping tools. All three trait prediction accuracy measures were compared (MAE, RMSE, and Pearson’s correlation coefficient). The Bartlett test was used to test for equal variance.

with(MAE_data, bartlett.test(MAE ~ Tool))

The p-values for all Bartlett tests were above 0.05 and therefore we did not reject the null hypothesis that group variances were all equal, for any accuracy measure. Example code for the ANOVA analyses is given below:

anova(lm(MAE ~ Tool, data = MAE_data))

For ANOVA analyses with a significant p-value – with a p-value threshold of 0.05 – pair-wise t-tests were conducted with a holm p‑value adjustment.

with(MAE_data, pairwise.t.test(MAE, Tool, pool.sd = TRUE))

S3.2 Results and Discussion

S3.2.1 Haplotype tool comparison

Many algorithms have been developed to estimate haplotypes; however, the best performing haplotype algorithm for GS is different for each species and trait combination (Difabachew *et al*., 2023; Weber *et al*., 2023). Some commonly used haplotyping tools are PLINK (Purcell *et al*. 2007, <https://zzz.bwh.harvard.edu/plink/ld.shtml>) and HaploBlocker (Pook *et al*., 2019). PLINK uses Gabriel’s haplotyping algorithm, which has been found to outperform other LD-based haplotyping methods (Weber *et al*., 2023). HaploBlocker is a haplotyping tool that uses linkage to define haplotype blocks (Pook *et al*., 2019), defined as regions of markers with a predefined frequency within the population. Each of these tools output the haplotype boundaries and their SNPs, but they do not identify haplotype variants or format the output for GWAS or GS analyses. The GS studies that use these haplotyping tools either choose a representative SNP from each haplotype block – randomly or based on the highest variance – or use custom code to identify haplotype variants (Difabachew *et al*., 2023; He *et al*., 2023; Ma *et al*., 2016; Marquez *et al*., 2024; Matias *et al*., 2017). Supplemental Table 7 shows a comparison between PLINK, HaploBlocker, and HaploVar. While HaploVar can identify haplotype variants and formats the output for GWAS and GS analyses, this comes at the cost of computational efficiency, especially for large datasets. Each haplotyping tool found a different number of haplotypes, with different average lengths. PLINK had the largest number of haplotypes due to their small size. The custom code applied to the PLINK haplotypes identified the highest number of haplotype variants. On the other hand, HaploVar found the largest and therefore the fewest haplotypes. While HaploBlocker identified smaller haplotypes than HaploVar, these haplotypes had a higher average number of variants as intra-haplotype outliers are not removed.

**Supplemental Table 7.** Comparison of the haplotyping tools HaploVar, PLINK (Purcell et al. 2007, https://zzz.bwh.harvard.edu/plink/ld.shtml), and HaploBlocker (Pook et al., 2019). Time and memory were measured using the sacct command for chromosome segment VCFs of sizes 60,518 KB to 807,219 KB. The number and length of haplotype and haplotype variants refer to all haplotypes within the A and C genomes in the Brassica napus data. Haplotype variants for HaploBlocker and PLINK were identified with custom code. The B. napus data published by Wu and colleagues (2019) and downloaded from CropGS-Hub (Chen et al., 2024, https://iagr.genomics.cn/CropGS/#/Datasets?species=Rapeseed).

|  | **PLINK** | **HaploBlocker** | **HaploVar** |
| --- | --- | --- | --- |
| Haplotyping algorithm | Gabriel’s algorithm (Gabriel *et al*., 2002) | HaploBlocker | DBSCAN (Ester *et al*. 1996) |
| Remove intra-haplotype outliers | No | No | Yes |
| Identify haplotype variants | No | No | Yes |
| Format output for GWAS and GS | No | No | Yes |
| Time | 3 sec to 8 sec | 28 min 23 sec to  1 hr 11 min 28 sec | 8 min 31 sec to 10 hrs 19 sec |
| Memory | 44.48 MB to 52.71 MB | 2.35 GB to 5.50GB | 8.27 GB to 48.19 GB |
| Number of haplotypes | 451,245 | 108,762 | 820 |
| Number of haplotype variants | 1,464,742 | 1,373,836 | 2,262 |
| Average number of variants per haplotype | 3 | 13 | 3 |
| Average length of haplotypes (bp) | 114 | 17,216 | 37,278 |

### S3.2.2 Genome-wide association studies

Haplotype variant-based GWAS results for the haplotyping tools PLINK, HaploBlocker, and HaploVar are displayed in Supplemental Table 8. The HaploVar-based GWAS was the most efficient, running at approximately 1 minute and 240 MB while PLINK and HaploBlocker ran for 32 to 34 minutes using 1.3 to 1.4 GB of memory. The highest number of significant genotype variants were found for HaploBlocker-based GWAS. On the other hand, a higher percent of HaploVar haplotype variants were significant indicating that they are more likely to be functionally important.

**Supplemental Table 8.** Genome-wide association studies (GWAS) results for flowering time in Brassica napus using haplotype variants identified using HaploVar, PLINK (Purcell et al. 2007, https://zzz.bwh.harvard.edu/plink/ld.shtml), HaploBlocker (Pook et al., 2019) and custom code. GWAS analysis was done using GEMMA and the parameter -maf 0 (Zhou and Stephens, 2012). The p-values were corrected using false discovery rate (FDR) (Benjamini and Hochberg 1995). The significance threshold was set to a p-value of 0.05. Time and memory used for the GWAS analyses were measured using the sacct command. The B. napus data published by Wu and colleagues (2019) and downloaded from CropGS-Hub (Chen et al., 2024, https://iagr.genomics.cn/CropGS/#/Datasets?species=Rapeseed).

|  | **PLINK** | **HaploBlocker** | **HaploVar** |
| --- | --- | --- | --- |
| Number of significant genetic variants | 91 | 143 | 1 |
| Percent of significant genetic variants (%) | 0.006 | 0.010 | 0.044 |
| Time | 33 min 54 sec | 32 min 47 sec | 1 min 2 sec |
| Memory | 1.39 GB | 1.32 GB | 240.36 MB |

### S3.2.3 Genomic selection

Haplotype variant-based GS results for the haplotyping tools PLINK, HaploBlocker, and HaploVar are displayed in Supplemental Table 9. PLINK-based GS had the highest prediction accuracy with the highest Pearson’s correlation coefficient score and the lowest errors. The Pearson’s correlation coefficient was significantly higher, and the MAE was significantly lower than the corresponding accuracy measures for HaploVar and HaploBlocker. The pair-wise t-tests comparing PLINK to HaploVar had a p-value of <0.001 and 0.008 for Pearson’s correlation coefficient and MAE, respectively. The pair-wise t‑tests between PLINK and HaploBlocker calculated p-values of 0.003 for Pearson’s correlation coefficient and 0.007 for MAE. RMSE was not found to be significantly different between any of the three haplotyping tools. The ANOVA for RMSE calculated a p-value of 0.065. The lower trait prediction accuracy for HaploVar and HaploBlocker may be due to the high number of variants per haplotype. Increasing levels of multiallelism increase multicollinearity, which has been found to reduce trait prediction accuracy in haplotype‑based GS (Hess et al., 2017; Sallam et al., 2020). This could be mitigated with a higher minFreq value. While HaploVar did not outperform PLINK or HaploBlocker in trait prediction accuracy, it was much more computationally efficient.

**Supplemental Table 9.** Genomic selection (GS) results for flowering time in Brassica napus using haplotype variants identified using HaploVar, PLINK (Purcell et al. 2007, https://zzz.bwh.harvard.edu/plink/ld.shtml), HaploBlocker (Pook et al., 2019) and custom code. GS was run using the model genomic best linear unbiased prediction (GBLUP) and the package rrBLUP (Endelman, 2011). Time and memory used while running the GS models were measured using the sacct command. The B. napus data published by Wu and colleagues (2019) and downloaded from CropGS-Hub (Chen et al., 2024, https://iagr.genomics.cn/CropGS/#/Datasets?species=Rapeseed). SD is an abbreviation of standard deviation.

|  | **PLINK** | **HaploBlocker** | **HaploVar** |
| --- | --- | --- | --- |
| Average Pearson's correlation coefficient (SD) | 0.582 (0.076) | 0.504 (0.084) | 0.487 (0.065) |
| Average root mean squared error (SD) | 8.819 (0.718) | 9.292 (0.738) | 9.300 (0.726) |
| Average mean absolute error (SD) | 6.747 (0.352) | 7.159 (0.330) | 7.064 (0.321) |
| Average time | 11 hr 19 min 55 sec | 2 days 4 hrs 11 min | 7.2 sec |
| Average memory | 58.51 GB | 55.88 GB | 863.2 KB |

S3.3 Conclusion

HaploVar includes additional features compared to other haplotyping tools including removing intra-haplotype outliers, identifying haplotype variants, and formatting the output for GWAS and GS analyses. These additional features come at the cost of computational efficiency, especially for large datasets. HaploVar can improve the efficiency of GWAS and GS analyses compared to other haplotyping tools and algorithms. HaploVar is a novel haplotyping tool that has the potential to streamline GWAS and GS pipelines.

# Supplementary Note 4: Parameter sensitivity analysis

In this section we conduct a parameter sensitivity analysis to show how the main HaploVar parameters affect the resulting haplotype statistics.

S4.1 Methods

See Supplementary Note 3 section S3.1.1 SNP preparation, for the methods used to prepare the SNPs for haplotype analysis. All following analysis were done using the A genome of the *B. napus* data. Haplotypes for all chromosome segment and corresponding linkage disequilibrium matrices were calculated and collated using HaploVar’s haplotype_variants and collate_haplotype_variants functions, respectively. Haplotypes for the epsilon values 0.1, 0.2, 0.3, 0.4, 0.5, 0.6, 0.7, 0.8, 0.9, and 1 were calculated with the format set to six and all other HaploVar parameters set to default. To test the sensitivity of MGmin, the HaploVar parameters epsilon and format were set to 0.7 and 6, respectively. Haplotypes were calculated for the MGmin values 5, 10, 15, 20, 25, 30, 35, and 40. The values 1, 2, 3, 4, 5, 6, 7, 8, 9, and 10 were used to test the sensitivity of the HaploVar’s minFreq parameter, with the parameters epsilon = 0.7, MGmin = 10, and format = 6.

S4.2 Results and Discussion

The general trends of how HaploVar parameters influence the resulting haplotypes are expected to be consistent across datasets; however, the optimal parameters will differ between species and datasets based on their underlying genetic architecture, linkage disequilibrium patterns, and genetic diversity. For example, a GS study in rice and maize found that rice had a higher diversity and greater frequency of rare haplotype variants than maize, which corresponded with lower trait prediction accuracy (Matias *et al.,* 2017). Therefore, the rice dataset would have a greater benefit from a higher minFreq value than the maize dataset. DBSCAN-based clustering in soybean found the optimal epsilon value to be 0.2 (Mohamedikbal et al., 2024), while no haplotypes were found for this epsilon value in the *B. napus* dataset used in this study.

The results for the parameter sensitivity analysis are shown in Supplemental Figures 3-5. Increasing epsilon value increased the number of haplotypes, number of haplotype variants, and average number of variants per haplotype (Supplementary Figure 3). The average haplotype length had an overall upward trend with increasing epsilon, although it fluctuated between epsilon values. The number of haplotypes, variants and variants per haplotype decrease with increasing MGmin, while the average haplotype length increases (Supplementary Figure 4). Increasing minFreq decreases the number of haplotype variants and variants per haplotype but does not affect the number of haplotypes or the haplotype length (Supplementary Figure 5).

| 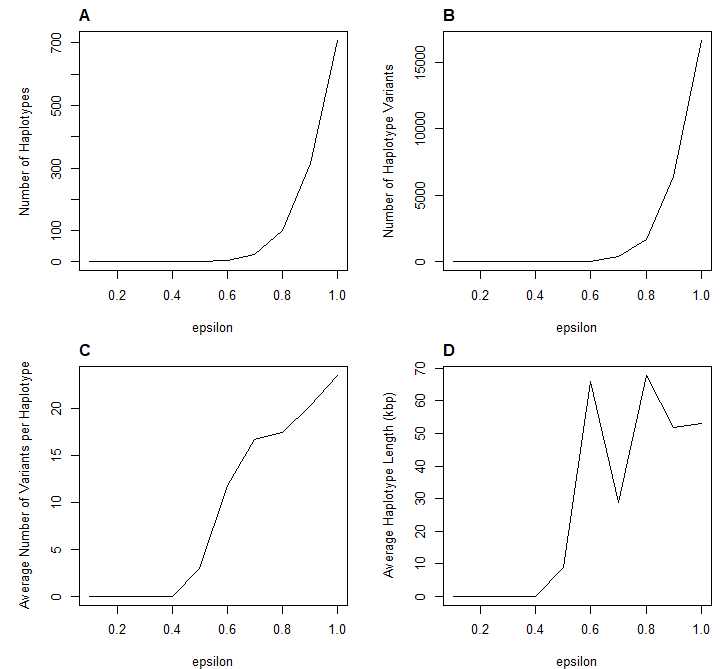 |
| --- |

**Supplemental Figure 3.** Haplotype statistics for a range of epsilon values (0.1, 0.2, 0.3, 0.4, 0.5, 0.6, 0.7, 0.8, 0.9, and 1) for the A genome of Brassica napus data published by Wu and colleagues (2019) and downloaded from CropGS-Hub (Chen et al., 2024, https://iagr.genomics.cn/CropGS/#/Datasets?species=Rapeseed). Haplotype variants were calculated by HaploVar’s haplotype_variants function with format set to 6 and all other parameters set to default. The haplotype statistic displayed are A) the number of haplotypes, B) the number of haplotype variants, C) the average number of variants per haplotype, and D) the average haplotype length in kilobase pairs (kbp).

| 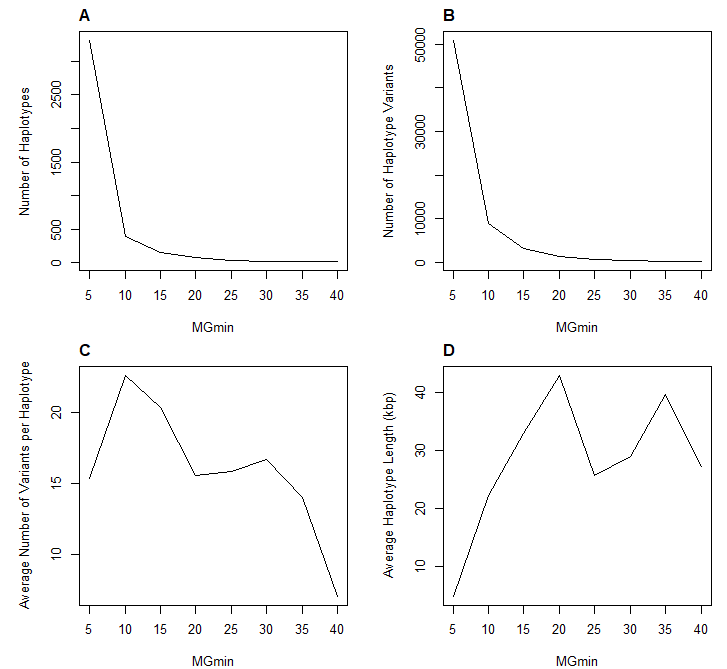 |
| --- |

**Supplemental Figure 4.** Haplotype statistics for a range of MGmin values (5, 10, 15, 20, 25, 30, 35, and 40) for the A genome of Brassica napus data published by Wu and colleagues (2019) and downloaded from CropGS-Hub (Chen et al., 2024, https://iagr.genomics.cn/CropGS/#/Datasets?species=Rapeseed). Haplotype variants were calculated by HaploVar’s haplotype_variants function with format set to 6 and epsilon set to 0.7. The haplotype statistic displayed are A) the number of haplotypes, B) the number of haplotype variants, C) the average number of variants per haplotype, and D) the average haplotype length in kilobase pairs (kbp).

| 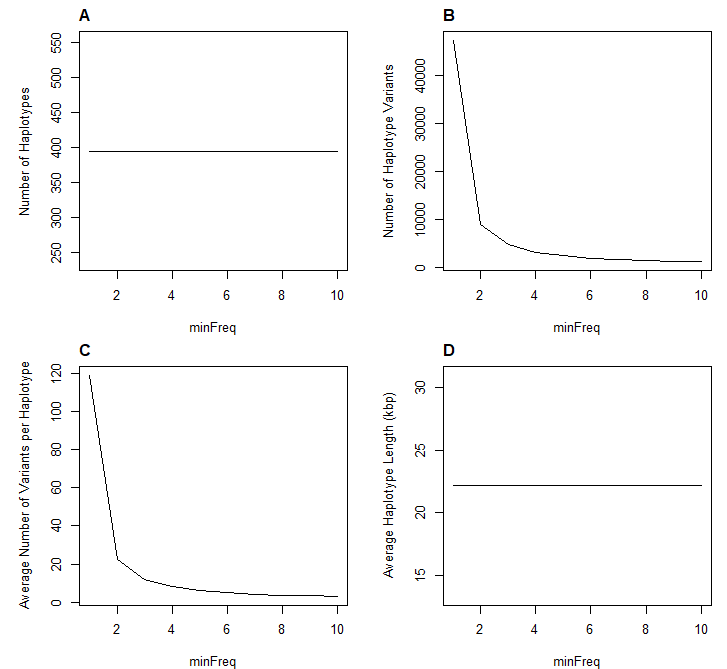 |
| --- |

**Supplemental Figure 5.** Haplotype statistics for a range of minFreq values (1, 2, 3, 4, 5, 6, 7, 8, 9, and 10) for the A genome of Brassica napus data published by Wu and colleagues (2019) and downloaded from CropGS-Hub (Chen et al., 2024, https://iagr.genomics.cn/CropGS/#/Datasets?species=Rapeseed). Haplotype variants were calculated by HaploVar’s haplotype_variants function with the parameters epsilon = 0.7, MGmin = 10, and format = 6. The haplotype statistic displayed are A) the number of haplotypes, B) the number of haplotype variants, C) the average number of variants per haplotype, and D) the average haplotype length in kilobase pairs (kbp).

## References

Abed A, Belzile F. Comparing single‐SNP, multi‐SNP, and haplotype‐based approaches in association studies for major traits in barley. *Plant Genome* 2019;12:1-14. https://doi.org/10.3835/plantgenome2019.05.003

Altschul SF, Gish W, Miller W *et al.* Basic local alignment search tool. *J Mol Biol* 1990;215:403–410. https://doi.org/10.1016/S0022-2836(05)80360-2

Bekele WA, Wight CP, Chao S *et al*. Haplotype‐based genotyping‐by‐sequencing in oat genome research. *Plant Biotechnol J* 2018;16:1452-1463. https://doi.org/10.1111/pbi.12888

Benjamini Y, Hochberg Y. Controlling the false discovery rate: A practical and powerful approach to multiple testing. *J R Stat Soc Series B Methodol* 1995;57:289–300. https://doi.org/10.1111/j.2517-6161.1995.tb02031

Browning BL, Zhou Y, Browning SR. A one-penny imputed genome from next-generation reference panels. *Am J Hum Genet* 2018;103:338–348. https://doi.org/10.1016/j.ajhg.2018.07.015

Browning BL, Tian X, Zhou Y *et al*. Fast two-stage phasing of large-scale sequence data. *Am J Hum Genet* 2021;108:1880–1890. https://doi.org/10.1016/j.ajhg.2021.08.005

Budhlakoti N, Mishra DC, Rai A *et al*. *_STGS: Genomic Selection using Single Trait_*. R package version 0.1.0. 2019. https://CRAN.R-project.org/package=STG

Chen J, Tan C, Zhu M *et al*. CropGS-Hub: a comprehensive database of genotype and phenotype resources for genomic prediction in major crops. *Nucleic Acids Res* 2024;52:D1519–D1529. https://doi.org/10.1093/nar/gkad1062

Clot CR, Klein D, Koopman J *et al*. Crossover shortage in potato is caused by StMSH4 mutant alleles and leads to either highly uniform unreduced pollen or sterility. *Genetics* 2024;226:Article iyad194. https://doi.org/10.1093/genetics/iyad194

Contreras-Soto RI, Mora F, De Oliveira *et al*. A genome-wide association study for agronomic traits in soybean using SNP markers and SNP-based haplotype analysis. *PloS One* 2017;12:e0171105-e0171105. https://doi.org/10.1371/journal.pone.0171105

Danecek P, Auton A, Abecasis G *et al*. The variant call format and VCFtools. *Bioinformatics* 2011;27:2156–2158. https://doi.org/10.1093/bioinformatics/btr33

Danecek P, Bonfield JK, Liddle J *et al*. Twelve years of SAMtools and BCFtools. *Gigascience* 2021;10:Article giab008. https://doi.org/10.1093/gigascience/giab008

Difabachew YF, Frisch M, Langstroff AL *et al*. Genomic prediction with haplotype blocks in wheat. *Front Plant Sci* 2023;14:Article 1168547. https://doi.org/10.3389/fpls.2023.1168547

Endelman JB. Ridge regression and other kernels for genomic selection with R package rrBLUP. *Plant Breed* 2011;4:250–255. https://doi.org/10.3835/plantgenome2011.08.0024

Ester M, Kriegel HP, Sander J *et al*. A density-based algorithm for discovering clusters in large spatial databases with noise. 1996.

Gabriel SB, Schaffner SF, Nguyen H *et al*. The structure of haplotype blocks in the human genome. *Science* 2002;296:2225–2229. https://doi.org/10.1126/science.1069424

Hamner B, Frasco M. *Metrics: Evaluation metrics for machine learning*. R package version 0.1.4. 2018. https://CRAN.R-project.org/package=Metrics

He S, Liang S, Meng L *et al*. Sparse phenotyping and haplotype-based models for genomic prediction in rice. *Rice* 2023;16:Article 27. https://doi.org/10.1186/s12284-023-00643-2

Helal M, Gill RA, Tang M *et al.* SNP- and haplotype-based GWAS of flowering-related traits in *Brassica napus*. *Plants* 2021;10:Article 2475. https://doi.org/10.3390/plants1011247

Hess M, Druet T, Hess A *et al*. Fixed-length haplotypes can improve genomic prediction accuracy in an admixed dairy cattle population. *Genet Sel Evol* 2017;49:1–14. https://doi.org/10.1186/s12711-017-0329-y

Khvorykh GV, Sapozhnikov NA, Limborska SA *et al*. Evaluation of density-based spatial clustering for Identifying genomic loci associated with ischemic stroke in genome-wide data. Int J Mol Sci 2023;24:Article 15355. https://doi.org/10.3390/ijms242015355

Kuhn M. Building predictive models in R using the caret package. *J Stat Softw* 2008;28:1–26. https://doi.org/10.18637/jss.v028.i05

Ma Y, Reif JC, Jiang Y *et al*. Potential of marker selection to increase prediction accuracy of genomic selection in soybean (*Glycine max* L.). *Mol Breed* 2016;36:Article 113. https://doi.org/10.1007/s11032-016-0504-9

Marquez GR, Zhang-Biehn S, Guo Z *et al*. Effects of marker density on genomic prediction for yield traits in sweet corn. *Euphytica* 2024;220:Article 52. https://doi.org/10.1007/s10681-024-03313-6

Matias FI, Galli G, Correia Granato IS *et al*. Genomic prediction of autogamous and allogamous plants by SNPs and haplotypes. *Crop Sci* 2017;57:2951–2958. https://doi.org/10.2135/cropsci2017.01.0022

Meena VK, Thribhuvan R, Dinkar V *et al*. Haplotype breeding: fast-track the crop improvements. *Planta* 2025;261:Article 51. https://doi.org/10.1007/s00425-025-04622-3

Mohamedikbal S, Al‐Mamun HA, Marsh JI *et al*. Local haplotyping reveals insights into the genetic control of flowering time variation in wild and domesticated soybean. *Plant Genome* 2024;17:Article e20528. https://doi.org/10.1002/tpg2.20528

Pook T, Schlather M, de los Campos G *et al*. HaploBlocker: Creation of subgroup-specific haplotype blocks and libraries. *Genetics* 2019;212:1045–1061. https://doi.org/10.1534/genetics.119.302283

Purcell S, Neale B, Todd-Brown K *et al.* PLINK: A tool set for whole-genome association and population-based linkage analyses. *Am J Hum Genet* 2007;81:559–575. https://doi.org/10.1086/519795

R Core Team. *_R: A language and environment for statistical computing_*. R Foundation for Statistical Computing, Vienna, Austria. 2024. https://www.R-project.org/

Sallam AH, Conley E, Prakapenka D *et al*. Improving prediction accuracy using multi-allelic haplotype prediction and training population optimization in wheat. *G3*  2020;10:2265–2273. https://doi.org/10.1534/g3.120.401165

Turner S. qqman: an R package for visualizing GWAS results using Q-Q and manhattan plots. *Journal of Open Source Software* 2018;3:731. https://doi.org/10.21105/joss.00731

Weber SE, Frisch M, Snowdon RJ *et al*. (2023). Haplotype blocks for genomic prediction: A comparative evaluation in multiple crop datasets. *Front Plant Sci* 2023;14:Article 1217589. https://doi.org/10.3389/fpls.2023.1217589

Wu D, Liang Z, Yan T *et al*. Whole-genome resequencing of a worldwide collection of rapeseed accessions reveals the genetic basis of ecotype divergence. *Mol Plant* 2019;12:30-43. https://doi.org/10.1016/j.molp.2018.11.007

Zhou X, Stephens M. Genome-wide efficient mixed-model analysis for association studies. *Nat Genet* 2012;44:821–824. https://doi.org/10.1038/ng.2310
